# Screening for Emotional Expression in Frontotemporal Dementia: A Pilot Study

**DOI:** 10.1155/2018/8187457

**Published:** 2018-03-01

**Authors:** Andrew R. Carr, Mark M. Ashla, Elvira E. Jimenez, Mario F. Mendez

**Affiliations:** ^1^VA Greater Los Angeles Healthcare System, Los Angeles, CA, USA; ^2^Department of Neurology, David Geffen School of Medicine, University of California at Los Angeles, Los Angeles, CA, USA; ^3^Department of Psychiatry & Biobehavioral Sciences, David Geffen School of Medicine, University of California at Los Angeles, Los Angeles, CA, USA

## Abstract

**Objective:**

Although emotional blunting is a core feature of behavioral variant frontotemporal dementia (bvFTD), there are no practical clinical measures of emotional expression for the early diagnosis of bvFTD.

**Method:**

Three age-matched groups (bvFTD, Alzheimer's disease (AD), and healthy controls (HC)) of eight participants each were presented with real-life vignettes varying in emotional intensity (high versus low) with either negative or positive outcomes. This study evaluated verbal (self-reports of distress) and visual (presence or absence of facial affect) measures of emotional expression during the vignettes.

**Results:**

The bvFTD patients did not differ from the AD and HC groups in reported distress or in the amount of facial affect during vignettes with high emotional intensity or type of outcome. However, the bvFTD patients reported significantly less distress and had correspondingly few facial affective expressions when compared on vignettes of low intensity.

**Conclusions:**

Patients with bvFTD require a high intensity of emotional stimulus and are significantly hyporesponsive to low-intensity stimuli. Simple screening or observations of verbal and facial responsiveness to mildly arousing stimuli may aid in differentiating bvFTD from normal subjects and patients with other dementias. Future studies can investigate whether delivering information with high emotional intensity can facilitate communication with patients with bvFTD.

## 1. Introduction

Behavioral variant frontotemporal dementia (bvFTD) results in socioemotional changes including abnormalities in context-appropriate behavior and personality [1]. These changes manifest as apathy or abulia, loss of empathy or sympathy, and disinhibition or poor impulse control, as well as other behaviors [2, 3]. Numerous studies demonstrate prominent impairments in socioemotional processing suggesting generalized emotional blunting may underlie many of these disturbed behaviors in bvFTD [4–6].

BvFTD patients generally fail to report distress and demonstrate decreased reactivity to emotional stimuli [7, 8]. Decreased verbal and facial expressions of emotion may result from an impaired ability to correctly recognize emotions [9–12], evident in decreased recognition of facial affect [9, 13–15]. Patients with bvFTD are especially impaired in the recognition of negative emotions [11, 16, 17], with relative preservation of the recognition of pleasant emotions such as happiness [10, 11, 15, 18]. Thus, while bvFTD patients demonstrate deficits in recognizing most emotions, and a striking paucity of emotional reactions [14, 19], they are not entirely devoid of emotional recognition.

There is a relationship of decreased emotional reactivity, manifest as decreased verbal and visual emotional expression, and decreased empathic behavior. The lack of verbal or visual emotional expression, operationalized as “emotional blunting” or “callous-unemotional” traits, is correlated not only with lack of empathy in bvFTD [4, 7] but also with decreased cognitive and affective empathy, affective perspective taking, and psychopathic traits in other populations [20, 21]. Empathy in bvFTD is a complex multidimensional construct and may correspond more directly to decreased emotional expressivity rather than with more traditional measures of emotion in bvFTD, such as the ability to identify facial expressions [22, 23]. To date, there has been little work on this relationship.

The lack of emotional expressivity may be a major clinical clue to the presence of bvFTD and, as such, could serve as the basis for a clinical test. Such a test is needed because the differentiating of bvFTD from other dementias, psychiatric conditions, and even normal subjects is important for management and prognosis [24, 25]. For example, there are patients with the behavioral criteria of bvFTD who prove to have Alzheimer's disease (AD) [26–28] and there are patients with bvFTD who have prominent memory deficits as in AD [29, 30]. In most clinical situations, there are no readily available and definitive biomarkers for screening these patients, again emphasizing the need for a practical screening test for bvFTD.

This study investigated screening for verbal and visual emotional expressions during video and audio presentation of real-life vignettes among patients with bvFTD compared with those with AD and healthy control (HC) subjects. Emotional expressions may vary depending on the emotional intensity of the eliciting stimuli [31, 32], and psychophysiological studies indicate a lowering of basic sympathetic tone in bvFTD, with reactivity to more intense stimuli [7]. Accordingly, this study compares self-reports of emotional distress and degree of facial expressivity on a standardized facial coding system during viewing of emotional vignettes of both high and low emotional intensity having either negative or positive outcomes.

## 2. Methods

### 2.1. Participants

Among patients in a university subspecialty clinic, this study recruited participants diagnosed with probable bvFTD per International Consensus Criteria [1], all of whom met criteria for loss of sympathy or empathy, and comparably impaired patients with clinically probable AD per National Institute on Aging–Alzheimer Association criteria [33]. The AD patients were age matched with the bvFTD patients within three years. Additionally, age-matched HC participants were recruited from volunteers in the community. Exclusion criteria included the presence of medical or psychiatric comorbidities save those of hypertension and diabetes. The dementia patients completed a screening interview, a standard neurological examination, and cognitive screening. The UCLA Institutional Review Board (IRB) approved this study, and each participant provided informed consent.

### 2.2. Procedures

The presentation of vignettes and the recording of facial affect were conducted in a quiet testing room. On entering the room, the participants were instructed to sit in a comfortable chair situated three feet in front of a computer screen and given instructions on the vignettes. They were asked to monitor the screen for a prompt indicating the onset of a vignette. During the vignette presentations, they were told to take note of their levels of distress. They were also informed that, during the viewing of the vignette, a video camera would be recording their visual gaze for purposes of assuring their attention to the vignettes and to later evaluate their facial responses. Finally, the participants were informed that their level of distress and recollection of the vignette would be queried immediately after each vignette.

#### 2.2.1. Video Vignettes

The study involved 16 emotional vignettes of 30 seconds or less duration presented in a randomized order in three different counterbalanced presentation sequences. All vignettes were performed by the same actor looking directly into the camera and presented through SuperLab Pro 4.0 with audio through headphones. There were four basic vignette scenarios: (1) relating the results of a test for cancer, (2) reporting on an automobile accident involving the presenter's grandfather, (3) informing on the status of a missing child, and (4) discussing the military deployment of the presenter's brother. These four scenarios were each presented with high intensity (delivered with prominent vocal prosody and facial affect) or low intensity (delivered with a flat and static voice and facial image) and with a final (last few seconds) negative outcome or a positive outcome, for a total of 16 vignettes. During development, an independent group of 10 HC participants analyzed the emotional intensity of these vignettes on a five-point scale, where “1”—not intense and “5”—very intense, and reliability agreed on emotional intensity content (*k* = 0.91). The items resulted in a bimodal distribution consistent with our classification of 8 “high-intensity” versus 8 “low-intensity” vignettes.

#### 2.2.2. Verbal Distress Ratings

Immediately after each vignette presentation, participants were queried on their comprehension of the vignettes and asked to rate their degree of distress on a Likert scale responding to the question, “How do you feel at the end of the story?” On the scale, “1” anchored to “no distress” and “5” anchored to “very distressed.” In order to assure understanding as well as attention to the vignettes, participants were also asked to describe each vignette's content in their own words and their responses recorded for the presence of understanding of the essence of each vignette.

#### 2.2.3. Facial Coding

Two independent raters coded the videos of the participants for facial expression using an established coding system for facial affect, the Facial Action Coding System (FACS) [34]. Both were FACS-certified raters who had previously practiced facial coding on a group of 7 normal subjects who watched the vignettes while being videotaped. For this study, the raters coded the participants' facial expressions (e.g., furrowed brow, angry) during the vignettes and determined whether they expressed any identifiable emotion, as defined by the FACS system. During viewing of each 30-second vignette, the raters coded each for the presence or absence of any observable facial expressions with “1” and no identifiable facial expressions with “0” with summary scores for the higher intensity and the lower intensity vignettes. The two raters demonstrated substantial interrater reliability, *k* = 0.80.

### 2.3. Statistical Analyses

This study utilized SPSS 23.0 software for statistical analysis. Chi-square and *F*-tests were used to evaluate group differences, and analyses of variance (ANOVA) were used for formal hypothesis testing. Pearson correlations assessed the relations between the distress scores and the number of facial expressions for each vignette. Post hoc analyses of pairwise comparisons were done with Fisher's least significant difference (LSD) and Tukey tests.

## 3. Results

### 3.1. Basic Group Comparisons

There were no significant group differences on demographic variables of age, sex, years of education, or handedness (see Table 1). The two dementia groups did not differ on the Montreal Cognitive Assessment scale [35].

### 3.2. Vignette General Results

The participants' attention and understanding of the vignettes were adequate for the study. On the videotapes, they maintained their eye gaze on the screen during the vignettes. After each vignette, all subjects demonstrated understanding of the vignettes by relating the essence of each vignette's content.

### 3.3. Reported Distress Levels

**Figure 1:**
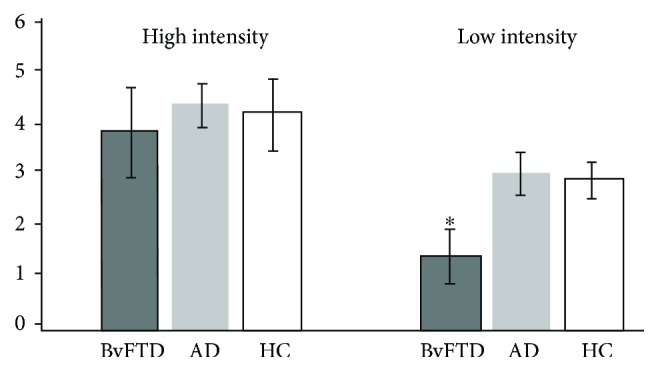
Group distress by level of vignette intensity among behavioral variant frontotemporal dementia (bvFTD), Alzheimer's disease (AD), and healthy control (HC) groups. The bvFTD group reported less distress (*M* = 1.40, SD = 1.05) than the other two groups when listening and viewing lower intensity vignettes (*M*_AD_ = 3.01, SD_AD_ = 0.95; *M*_HC_ = 2.88, SD_HC_ = 0.71). The groups reported no differences in the level of distress among higher intensity vignettes (*M*_bvFTD_ = 3.81, SD_bvFTD_ = 1.75; *M*_AD_ = 4.34, SD_AD_ = 0.95; *M*_HC_ = 4.15, SD_HC_ = 1.38). Asterisk indicates post hoc significance of the bvFTD group from the other groups.

The bvFTD group reported less distress than the other two groups when listening and viewing lower intensity vignettes (*η*^2^ = 0.41, *F*(2, 19) = 9.85, *p* = 0.003; see Figure 1). Post hoc analysis for lower intensity vignettes reviewed significant pairwise differences for the bvFTD group versus the AD group and for the bvFTD group versus the HC group (both LSD and Tukey *p* < 0.05) but not between the AD group and HC group. In contrast, the groups reported no differences in level of distress among higher intensity vignettes. Additionally, there were no significant group differences in reported distress between vignettes with negative (high or low intensity) or positive (high or low intensity) outcomes.

### 3.4. Facial Coding

**Figure 2:**
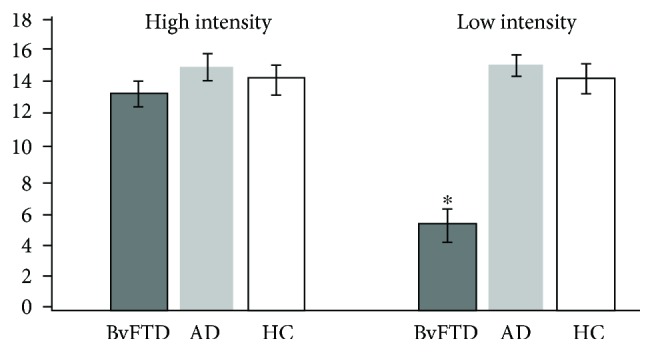
Group occurrences of facial affect by vignette intensity among behavioral variant frontotemporal dementia (bvFTD), Alzheimer's disease (AD), and healthy control (HC) groups. There were no differences in the presence of facial expression between the groups on the higher intensity vignettes (*M*_bvFTD_ = 13.13, SD_bvFTD_ = 1.61; *M*_AD_ = 15. 01, SD_AD_ = 1.66; *M*_HC_ = 14.20, *SD*_HC_ = 1. 79; *p* = 0.169), but the bvFTD patients demonstrated significantly fewer facial expressions on the lower intensity vignettes (*M*_bvFTD_ = 5.20, SD_bvFTD_ = 2.01; *M*_AD_ = 15.00, SD_AD_ = 1.25; *M*_HC_ = 14.20, *SD*_HC_ = 1.78). Asterisk indicates post hoc significance of the bvFTD group from the other groups.

Facial affect scores for the two raters were summed for the 8 higher intensity vignettes (*n* = 16) and the 8 lower intensity vignettes (*n* = 16). There were no differences in the presence of facial expression between groups on the higher intensity vignettes, but the bvFTD patients demonstrated significantly fewer facial expressions on the lower intensity vignettes (*η*^2^ = 0.49, *F*(2, 19) = 16.84, *p* < 0.001; see Figure 2). Post hoc analysis for lower intensity vignettes reviewed significant pairwise differences for the bvFTD group versus the AD group and for the bvFTD group versus the HC group (both LSD and Tukey *p* < 0.05) but not between the AD group and HC group. Similar to the verbal distress scores, there were no significant facial affect score differences between groups based on negative or positive outcomes.

### 3.5. Correlations between Self-Report Distress and Facial Affect

Across all three groups, the overall level of self-reported distress correlated with the total number of facial expressions across the vignettes (i.e., the participants' level of distress increased correspondingly to more facial affective expressions to the stimuli), *r*(24) = 0.76, *p* = 0.002. Within the bvFTD group, the total level of distress also correlated to the instances of facial affect, such that they frequently responded to high-intensity content with some type of facial expression but lacked responsiveness to low-intensity content, *r*(8) = 0.54, *p* = 0.021. Likewise, within the AD and HC groups, the reported distress level positively correlated with instances of facial affect (AD: *r*(8) = 0.62, *p* = 0.008; HC: *r*(8) = 0.77, *p* = 0.002).

## 4. Discussion

In this preliminary study, bvFTD and AD, the two most common early-onset neurodegenerative dementias, are easily distinguished on verbal and facial measures of emotional reactivity. This study examines emotional reactivity by comparing self-reported levels of distress and observing facial expressions to emotional vignettes of varying emotional intensity. When the vignettes were of high emotional intensity, the bvFTD patients did not differ from the AD and HC participants in self-reported level of distress or the amount of facial affect. However, when the emotional stimuli were of low intensity, the bvFTD participants significantly differed from the AD and HC participants, reporting less distress and showing fewer facial expressions of affect. Observing the lack of verbal and visual emotional responses to mild-moderate intensity, emotional stimuli in bvFTD suggests a promising method for distinguishing these patients from those with other dementias and from normal subjects.

The correct recognition and diagnosis of bvFTD is important for clinical management, clinical trial enrollment, genetic analysis, and the understanding of these disorders [24, 25]. Clinicians may misdiagnose bvFTD as AD if there are deficits in episodic declarative memory [29, 30] or if the patient does not meet established criteria for bvFTD [1]. In contrast, clinicians may misdiagnose AD as bvFTD in the presence of early neuropsychiatric features [27, 28] or in the presence of the frontal or “behavioral/dysexecutive” variant of AD [26]. Clinically accessible tests, such as positron emission tomography (PET) scans, could mitigate the number of misdiagnoses; however, even fluorodeoxyglucose PET scans can be normal in early bvFTD [36], and amyloid or tau PET imaging are not readily or economically available for AD.

The use of diagnostic measures of emotional expression in bvFTD is a good alternative, not only because of clinical availability but also because of the neuroanatomical foci of this disease. Compared to AD, where brain regions for emotional processing are relatively intact [37], bvFTD affects multiple neuroanatomical regions involved in emotion. Their lack of empathy and connectedness corresponds to atrophy of frontal regions, such as the anterior cingulate cortex (ACC) and ventromedial prefrontal cortex (VMPFC) [38, 39], and of the anterior temporal lobes [40, 41]. Symptoms of apathy and the tendency to respond to emotional situations with disinterest most often associate with frontal dysfunction, especially of the ACC and VMPFC [39, 42], and symptoms involving emotion processing, including emotional blunting and emotion recognition, additionally correlate with the right anterior temporal region [14]. Patients with bvFTD may fail to recognize or misinterpret emotional stimuli such as facial affect [10, 13, 43]. These patients may be unable to understand the context of the emotional stimuli, extract its meaning, experience interoceptive emotional awareness, or intentionally express emotions [44–48]. Furthermore, bvFTD patients have decreased physiological reactivity to emotional stimuli suggesting the need for a higher threshold of intensity to react to emotions [7].

In this study, the intensity of the emotional stimuli differentially affects the elicitation of self-reported distress and corresponding facial affect among the bvFTD participants compared to the other groups. Tasks varying in emotional intensity may be especially sensitive in detecting impairments in emotion recognition [49], regardless of the presence of a negative or a positive outcome. The results of this study suggests that bvFTD patients require a high intensity of emotional stimulus in order to elicit distress and affective expression. It is also possible that the relative preservation of happiness and surprise in some studies of bvFTD could indicate a greater experience of intensity for these emotions [10, 15]. In addition, in normal socioemotional settings, low levels of emotional intensity are the norm, so bvFTD patients may appear as if they have total affective blunting. Finally, the findings of this preliminary study suggest an approach to interactions and interventions for caregivers and others through the use of a higher intensity of emotional expression as a way to facilitate communication [20].

There are several methodological concerns with this study. First, the study has a relatively small sample size per group and may be underpowered. Nevertheless, in this pilot study, the number of participants per group is sufficient to disclose significant group differences. A future study with larger numbers can further investigate these findings. Second, the study used a Likert scale of distress, which only accounts for ratings from very upset to not very upset without assessing different individual emotions. Third, the lack of differential results based on negative or positive outcome could be based on the experimental design, as only the last few seconds of the vignette involved different outcomes. Finally, there could be concerns with the facial coding, and as with the verbal self-reports, this study did not specifically analyze the different types of emotions or the participants' recognition of specific emotions. This procedure, however, is an established technique for determining the presence or absence of facial affect, and this methodology lends itself to evaluation of specific emotions in future studies.

In summary, clinicians and investigators may observe and document the emotional blunting in bvFTD as a relative absence of verbal and facial emotional expression to mild-moderate emotional stimuli that routinely elicit emotional responses in others. Further systematic research on how to observe and record emotional expression in bvFTD, for example, as responses to simple picture stimuli in the clinic, may help better recognize, diagnose, and eventually manage these patients.

## Figures and Tables

**Table 1:** Characteristics between behavioral variant frontotemporal dementia (bvFTD), Alzheimer's disease (AD), and healthy control (HC) groups.

	BvFTD *M* (SD; range) or %	AD *M* (SD; range) or %	HC *M* (SD; range) or %	Sig.
Age (years)	61.3 (10.1; 42–78)	60.0 (4.9; 49–65)	59.0 (5.1; 49–66)	n.s.
Sex (% male)	50%	38%	38%	n.s.
Education (years)	15.9 (2.4; 12–20)	16.3 (2.0;12–18)	16.1 (3.1;12–18)	n.s.
Handedness (% right)	88%	88%	100%	n.s.
Montreal Cognitive Assessment	17.4 (6.3; 11–29)	16.9 (5.8; 11–23)	—	n.s.

*M* = mean; SD = standard deviation; % = percentage.

## References

[1] Rascovsky K., Hodges J. R., Knopman D. (2011). Sensitivity of revised diagnostic criteria for the behavioural variant of frontotemporal dementia. *Brain*.

[2] Mendez M. F., Shapira J. S. (2011). Loss of emotional insight in behavioral variant frontotemporal dementia or “frontal anosodiaphoria”. *Consciousness and Cognition*.

[3] Robertson D., Snarey J., Ousley O. (2007). The neural processing of moral sensitivity to issues of justice and care. *Neuropsychologia*.

[4] Joshi A., Barsuglia J. P., Mather M. J., Jimenez E. E., Shapira J., Mendez M. F. (2014). Evaluation of emotional blunting in behavioral variant frontotemporal dementia compared to Alzheimer’s disease. *Dementia and Geriatric Cognitive Disorders*.

[5] Lee G. J., Lu P. H., Mather M. J. (2014). Neuroanatomical correlates of emotional blunting in behavioral variant frontotemporal dementia and early-onset Alzheimer’s disease. *Journal of Alzheimer's Disease*.

[6] Mendez M. F., Fong S. S., Shapira J. S. (2014). Observation of social behavior in frontotemporal dementia. *American Journal of Alzheimer's Disease &Other Dementias*.

[7] Joshi A., Mendez M. F., Kaiser N., Jimenez E., Mather M., Shapira J. S. (2014). Skin conductance levels may reflect emotional blunting in behavioral variant frontotemporal dementia. *The Journal of Neuropsychiatry and Clinical Neurosciences*.

[8] Fletcher P. D., Nicholas J. M., Shakespeare T. J. (2015). Dementias show differential physiological responses to salient sounds. *Frontiers in Behavioral Neuroscience*.

[9] Kumfor F., Irish M., Hodges J. R., Piguet O. (2013). Discrete neural correlates for the recognition of negative emotions: insights from frontotemporal dementia. *PLoS One*.

[10] Lough S., Kipps C. M., Treise C., Watson P., Blair J. R., Hodges J. R. (2006). Social reasoning, emotion and empathy in frontotemporal dementia. *Neuropsychologia*.

[11] Lavenu I., Pasquier F., Lebert F., Petit H., der Linden M. V. (1999). Perception of emotion in frontotemporal dementia and Alzheimer disease. *Alzheimer Disease & Associated Disorders*.

[12] Rosen H. J., Pace-Savitsky K., Perry R. J., Kramer J. H., Miller B. L., Levenson R. W. (2004). Recognition of emotion in the frontal and temporal variants of frontotemporal dementia. *Dementia and Geriatric Cognitive Disorders*.

[13] Rosen H. J., Allison S. C., Schauer G. F., Gorno-Tempini M. L., Weiner M. W., Miller B. L. (2005). Neuroanatomical correlates of behavioural disorders in dementia. *Brain*.

[14] Kipps C. M., Nestor P. J., Acosta-Cabronero J., Arnold R., Hodges J. R. (2009). Understanding social dysfunction in the behavioural variant of frontotemporal dementia: the role of emotion and sarcasm processing. *Brain*.

[15] Kumfor F., Miller L., Lah S. (2011). Are you really angry? The effect of intensity on facial emotion recognition in frontotemporal dementia. *Social Neuroscience*.

[16] Bediou B., Ryff I., Mercier B. (2009). Impaired social cognition in mild Alzheimer disease. *Journal of Geriatric Psychiatry and Neurology*.

[17] Keane J., Calder A. J., Hodges J. R., Young A. W. (2002). Face and emotion processing in frontal variant frontotemporal dementia. *Neuropsychologia*.

[18] Fernandez-Duque D., Black S. E. (2005). Impaired recognition of negative facial emotions in patients with frontotemporal dementia. *Neuropsychologia*.

[19] Sturm V. E., Levenson R. W. (2011). Alexithymia in neurodegenerative disease. *Neurocase*.

[20] Lui J. H. L., Barry C. T., Sacco D. F. (2016). Callous-unemotional traits and empathy deficits: mediating effects of affective perspective-taking and facial emotion recognition. *Cognition and Emotion*.

[21] Kahn R. E., Byrd A. L., Pardini D. A. (2013). Callous-unemotional traits robustly predict future criminal offending in young men. *Law and Human Behavior*.

[22] Miller L. A., Hsieh S., Lah S., Savage S., Hodges J. R., Piguet O. (2012). One size does not fit all: face emotion processing impairments in semantic dementia, behavioural-variant frontotemporal dementia and Alzheimer’s disease are mediated by distinct cognitive deficits. *Behavioural Neurology*.

[23] Diehlschmid J., Pohl C., Ruprecht C., Wagenpfeil S., Foerstl H., Kurz A. (2007). The Ekman 60 Faces Test as a diagnostic instrument in frontotemporal dementia. *Archives of Clinical Neuropsychology*.

[24] Piguet O., Hornberger M., Mioshi E., Hodges J. R. (2011). Behavioural-variant frontotemporal dementia: diagnosis, clinical staging, and management. *The Lancet Neurology*.

[25] Mendez M. F., Shapira J. S., McMurtray A., Licht E. (2007). Preliminary findings: behavioral worsening on donepezil in patients with frontotemporal dementia. *The American Journal of Geriatric Psychiatry*.

[26] Ossenkoppele R., Pijnenburg Y. A. L., Perry D. C. (2015). The behavioural/dysexecutive variant of Alzheimer’s disease: clinical, neuroimaging and pathological features. *Brain*.

[27] Mendez M. F., Joshi A., Tassniyom K., Teng E., Shapira J. S. (2013). Clinicopathologic differences among patients with behavioral variant frontotemporal dementia. *Neurology*.

[28] Querfurth H. W., LaFerla F. M. (2010). Alzheimer’s disease. *The New England Journal of Medicine*.

[29] Hornberger M., Wong S., Tan R. (2012). *In vivo* and post-mortem memory circuit integrity in frontotemporal dementia and Alzheimer’s disease. *Brain*.

[30] Pleizier C. M., van der Vlies A. E., Koedam E. (2012). Episodic memory and the medial temporal lobe: not all it seems. Evidence from the temporal variants of frontotemporal dementia. *Journal of Neurology, Neurosurgery, & Psychiatry*.

[31] Baez S., Manes F., Huepe D. (2014). Primary empathy deficits in frontotemporal dementia. *Frontiers in Aging Neuroscience*.

[32] Levenson R. W., Miller B. L. (2007). Loss of cells—loss of self: frontotemporal lobar degeneration and human emotion. *Current Directions in Psychological Science*.

[33] McKhann G. M., Knopman D. S., Chertkow H. (2011). The diagnosis of dementia due to Alzheimer’s disease: recommendations from the National Institute on Aging-Alzheimer’s Association workgroups on diagnostic guidelines for Alzheimer’s disease. *Alzheimer's & Dementia*.

[34] Buck R. (1990). Using fags vs. communication scores to measure spontaneous facial expression of emotion in brain-damaged patients: a reply to Mammucari et al. (1988). *Cortex*.

[35] Nasreddine Z. S., Phillips N., Chertkow H. (2012). Normative data for the Montreal Cognitive Assessment (MoCA) in a population-based sample. *Neurology*.

[36] Kerklaan B. J., van Berckel B. N. M., Herholz K. (2014). The added value of 18-fluorodeoxyglucose-positron emission tomography in the diagnosis of the behavioral variant of frontotemporal dementia. *American Journal of Alzheimer's Disease & Other Dementias*.

[37] Karas G. B., Burton E. J., Rombouts S. A. R. B. (2003). A comprehensive study of gray matter loss in patients with Alzheimer’s disease using optimized voxel-based morphometry. *NeuroImage*.

[38] Eslinger P. J., Damasio A. R. (1985). Severe disturbance of higher cognition after bilateral frontal lobe ablation Patient EVR. *Neurology*.

[39] Peters F., Perani D., Herholz K. (2006). Orbitofrontal dysfunction related to both apathy and disinhibition in frontotemporal dementia. *Dementia and Geriatric Cognitive Disorders*.

[40] Brambati S. M., Renda N. C., Rankin K. P. (2007). A tensor based morphometry study of longitudinal gray matter contraction in FTD. *NeuroImage*.

[41] Rosen H. J., Kramer J. H., Gorno-Tempini M. L., Schuff N., Weiner M., Miller B. L. (2002). Patterns of cerebral atrophy in primary progressive aphasia. *The American Journal of Geriatric Psychiatry*.

[42] Liu W., Miller B. L., Kramer J. H. (2004). Behavioral disorders in the frontal and temporal variants of frontotemporal dementia. *Neurology*.

[43] Kumfor F., Irish M., Leyton C. (2014). Tracking the progression of social cognition in neurodegenerative disorders. *Journal of Neurology, Neurosurgery, & Psychiatry*.

[44] Carr A. R., Paholpak P., Daianu M. (2015). An investigation of care-based vs. rule-based morality in frontotemporal dementia, Alzheimer’s disease, and healthy controls. *Neuropsychologia*.

[45] Mendez M. F., Chen A. K., Shapira J. S., Miller B. L. (2005). Acquired sociopathy and frontotemporal dementia. *Dementia and Geriatric Cognitive Disorders*.

[46] Gola K. A., Shany-Ur T., Pressman P. (2017). A neural network underlying intentional emotional facial expression in neurodegenerative disease. *NeuroImage: Clinical*.

[47] Ibanez A., Gleichgerrcht E., Manes F. (2010). Clinical effects of insular damage in humans. *Brain Structure and Function*.

[48] Ibáñez A., Velásquez M. M., Caro M. M., Manes F. (2013). Implicit emotional awareness in frontotemporal dementia. *Cognitive Neuroscience*.

[49] Chiu I., Piguet O., Diehl-Schmid J. (2016). Dissociation in rating negative facial emotions between behavioral variant frontotemporal dementia and major depressive disorder. *The American Journal of Geriatric Psychiatry*.

